# Exploring the Characteristics and Preferences for Online Support Groups: Mixed Method Study

**DOI:** 10.2196/15987

**Published:** 2019-12-03

**Authors:** Melanie Louise Plinsinga, Manuela Besomi, Liam Maclachlan, Luciano Melo, Sarah Robbins, Belinda J Lawford, Pek Ling Teo, Kathryn Mills, Jenny Setchell, Thorlene Egerton, Jillian Eyles, Leanne Hall, Rebecca Mellor, David J Hunter, Paul Hodges, Bill Vicenzino, Kim Bennell

**Affiliations:** 1 School of Health and Rehabilitation Sciences The University of Queensland Brisbane Australia; 2 The Sax Institute Ultimo Australia; 3 Kolling Institute of Medical Research Institute of Bone and Joint Research The University of Sydney Sydney Australia; 4 Department of Rheumatology, Royal North Shore Hospital and Northern Clinical School Faculty of Medicine and Health The University of Sydney Sydney Australia; 5 Centre for Health, Exercise, and Sports Medicine Department of Physiotherapy University of Melbourne Carlton Australia; 6 Faculty of Medicine and Health Sciences Macquarie University Sydney Australia

**Keywords:** osteoarthritis, self-help groups, self-management, surveys and questionnaires

## Abstract

**Background:**

Osteoarthritis (OA) is a chronic, disabling, and prevalent disorder. As there is no cure for OA, long-term self-management is paramount. Support groups (SGs) can facilitate self-management among people living with OA. Understanding preferences in design and features of SGs, including online SGs (OSGs), among people with OA can inform future development of SG interventions for this condition.

**Objective:**

The objective of this study was to investigate health care– and health information–seeking behavior, digital literacy, and preferences for the design of SGs in people with OA. The study also explored the perceived barriers and enablers to being involved in OSGs.

**Methods:**

An online survey study was conducted with a mixed method design (quantitative and qualitative). Individuals aged ≥45 years with knee, hip, or back pain for ≥3 months were recruited from an extant patient database of the Institute of Bone and Joint Research via email invitations. Quantitative elements of the survey included questions about sociodemographic background; health care– and health information–seeking behavior; digital literacy; and previous participation in, and preferences for, SGs and OSGs. Respondents were classified into 2 groups (Yes-SG and No-SG) based on previous participation or interest in an SG. Group differences were assessed with Chi-square tests (significance level set at 5%). Responses to free-text questions relating to preferences regarding OSG engagement were analyzed qualitatively using an inductive thematic analysis.

**Results:**

A total of 415 people with OA completed the survey (300/415, 72.3% females; 252/415, 61.0% lived in a major city). The Yes-SG group included 307 (307/415, 73.9%) participants. Between the Yes-SG and No-SG groups, there were no differences in sociodemographic characteristics, health care– and health information–seeking behavior, and digital literacy. An online format was preferred by 126/259 (48.7%) of the Yes-SG group. Trained peer facilitators were preferred, and trustworthiness of advice and information were highly prioritized by the respondents. Qualitative analysis for OSG participation revealed 5 main themes. Lack of time and motivation were the main barriers identified. The main enablers were related to accessibility, enjoyment of the experience, and the content of the discussed information.

**Conclusions:**

These findings highlight the preferences in design features and content of SGs and OSGs and may assist in the further development of such groups.

## Introduction

Osteoarthritis (OA) is a highly prevalent chronic condition [1] and can have a significant negative impact on both the individual and society. OA is one of the leading causes of functional limitation in older adults [1] and is associated with considerable direct and indirect health care costs [2,3]. These costs are predicted to rise substantially over the coming decades [2]. Accessible, high-quality strategies that support people to self-manage OA successfully are urgently needed.

The international chronic condition self-management support (CCSMS) framework describes principles to guide the implementation of strategies to support self-management [4]. These principles recommend that strategies should be as follows: (1) informed by evidence and the needs of the users, (2) person-centered, (3) easily accessible, (4) offering choice and autonomy, (5) aligned with treatment options that are available, and (6) emphasizing maximum benefits while minimizing harms [4]. Self-management support strategies offered to people with knee and hip OA, as well as back pain, typically involve therapeutic exercise programs, general physical activity promotion, and weight loss programs for those who are overweight [5,6]. These OA self-management support strategies aim to reduce pain and improve physical function and the quality of life. Previous research into painful musculoskeletal disorders has shown that self-management support strategies that provide social support and networks may also lead to improved pain and self-efficacy and increase physical function [7-9]. Social support provided in groups promotes a sense of belonging and active interaction [10,11], something that is important for both the individual and the group—the individual must continue to participate to receive all of their benefits, and the group relies on the aggregate knowledge where a larger community is likely to know more about a problem than a smaller one [12]. Therefore, the addition of social support and networks could potentially improve the outcomes of people living with knee, hip, and back OA.

A medium through which people with OA can potentially access social support and networks is support groups (SGs). SGs aim to provide avenues for people with a disease or condition to share information, provide empathy, and promote positive health behaviors. Given the availability of the internet in most households in the Western countries [13,14] and the data showing an increase in online health service usage [14], online SGs (OSGs) may be an inexpensive and convenient way for people to participate in SGs. The number of OSGs has increased in recent years, particularly as adjuncts to traditional care [15]. The nature of such groups varies widely. A systematic review of SGs across all health conditions [16] reported that about half were found to include only peer-to-peer engagement, whereas the other half included peer-to-peer engagement as part of a multifactorial intervention. The latter may be moderated by health professionals or administrators [17]. How people engage in OSGs varies. Broadly, participants might be readers or posters. Among the posters, participants may be initiators, responders, authorities, discussants, supporters, and more. Many participant styles are unique to the health condition [18]. Retrospective studies suggest that the benefits obtained from participation may be influenced by how an individual chooses to participate, but direct associations are yet to be made [12,19]. Reducing depressive symptoms and improving social support are the most commonly proposed mechanisms by which the OSGs were thought to afford health benefits [18,20]. Other outcomes of interest include general well-being, empowerment, anxiety, quality of life, health care utilization, or specific behavior changes (eg, weight loss) [21-24].

If SGs and OSGs are to be employed as strategies of self-management support, the principles of the CCSMS framework should be considered [4]. However, currently, the first guiding principle for self-management support strategies (ie, informed by evidence and the needs of the users) cannot be met as there is a paucity of evidence to inform the design and implementation of effective OSGs, particularly, in relation to people with OA. No previous studies have investigated the needs and preferences of people with OA regarding the design features and content of OSGs. There is no evidence outlining whether people are willing to engage with such groups and reasons why or why not. We are also uninformed regarding the demographic profile of those who are willing to engage with SGs, compared with those who are not. This study surveyed people with OA to determine the needs of potential SG and OSG users by investigating the health care– and health information–seeking behavior, digital literacy, and preferences for the design of SGs. The specific study aims were as follows: (1) compare sociodemographic characteristics, health care– and health information–seeking behavior, and digital literacy between those who are currently using or interested in joining and those who are not using or not interested in joining SGs; (2) evaluate preferences for content, delivery method, and types of engagement in relation to SGs; and (3) explore the perceived barriers and enablers to being involved in OSGs.

## Methods

### Study Design and Setting

An online survey study was conducted with a mixed method design conforming with the checklist for reporting result of internet electronic surveys (Multimedia Appendix 1). Potentially eligible participants were identified from the patient database of the Institute of Bone and Joint Research (University of Sydney). An email invitation to participate, including a link to the survey, was sent to people who had consented to be contacted for future research opportunities. Ethics approval was obtained from the Human Research Ethics Committee (HREC) of the University of Sydney (HREC #2017/957). Online informed consent was provided before the survey could be accessed by clicking a required checkbox.

### Participants

People aged ≥45 years who had previously received a clinical diagnosis of OA for any joint [25] were invited to participate. The survey commenced with 2 screening questions: (1) “Are you over 45 years of age?” and (2) “Do you have knee, hip, or back pain lasting more than 3 months?” Respondents who answered *no* to either question were excluded from the survey. People with comorbidities (eg, diabetes and heart disease) were also eligible; however, questions pertaining to SGs were specific to musculoskeletal conditions.

### Sample Size

A generic sample size calculation was used to determine the minimum sample size needed for generalizable results, given the exploratory aims of the study. Considering the estimated population size of people living with OA (primarily affecting the hands, spine, knees, and hips) in Australia is over 2 million [26], an acceptable margin of error of 5%, and accepted confidence level of 95%, the minimum sample size required was 385.

### Procedure

Data collection occurred between March and September 2018. The survey was administered through the Research Electronic Data Capture (REDCap) survey software (version 9.3.6, Vanderbilt University) and comprised closed, open, and multiple-choice questions (Multimedia Appendix 2). The quantitative information (closed and multiple-choice questions) was collected across 4 blocks of questions: (1) sociodemographic characteristics, (2) health care– and health information–seeking behavior, (3) use of technology (digital literacy), and (4) participation and preferences of SGs. The specific musculoskeletal condition (ie, hip OA, knee OA or back pain) was not identified. The type and wording of each question was composed by the research team. Face validity was ascertained by asking a sample of patient representatives (N=5) to view and provide feedback on each question in blocks 2 to 4. The order of questions was not randomized. Rather, the survey followed a predetermined logic where contingent questions were included/skipped based on participants’ previous responses. Qualitative data were collected with 3 open questions exploring possible barriers and enablers to OSG engagement: “What would make it difficult for you to use an OSG?” (Q36), “What would make it easier for you to use an OSG?” (Q37), and “Is there anything else you would like to say about using OSGs?” (Q38). Recruitment and data collection were conducted concurrently.

### Analysis

#### Quantitative Analysis

**Figure 1:**
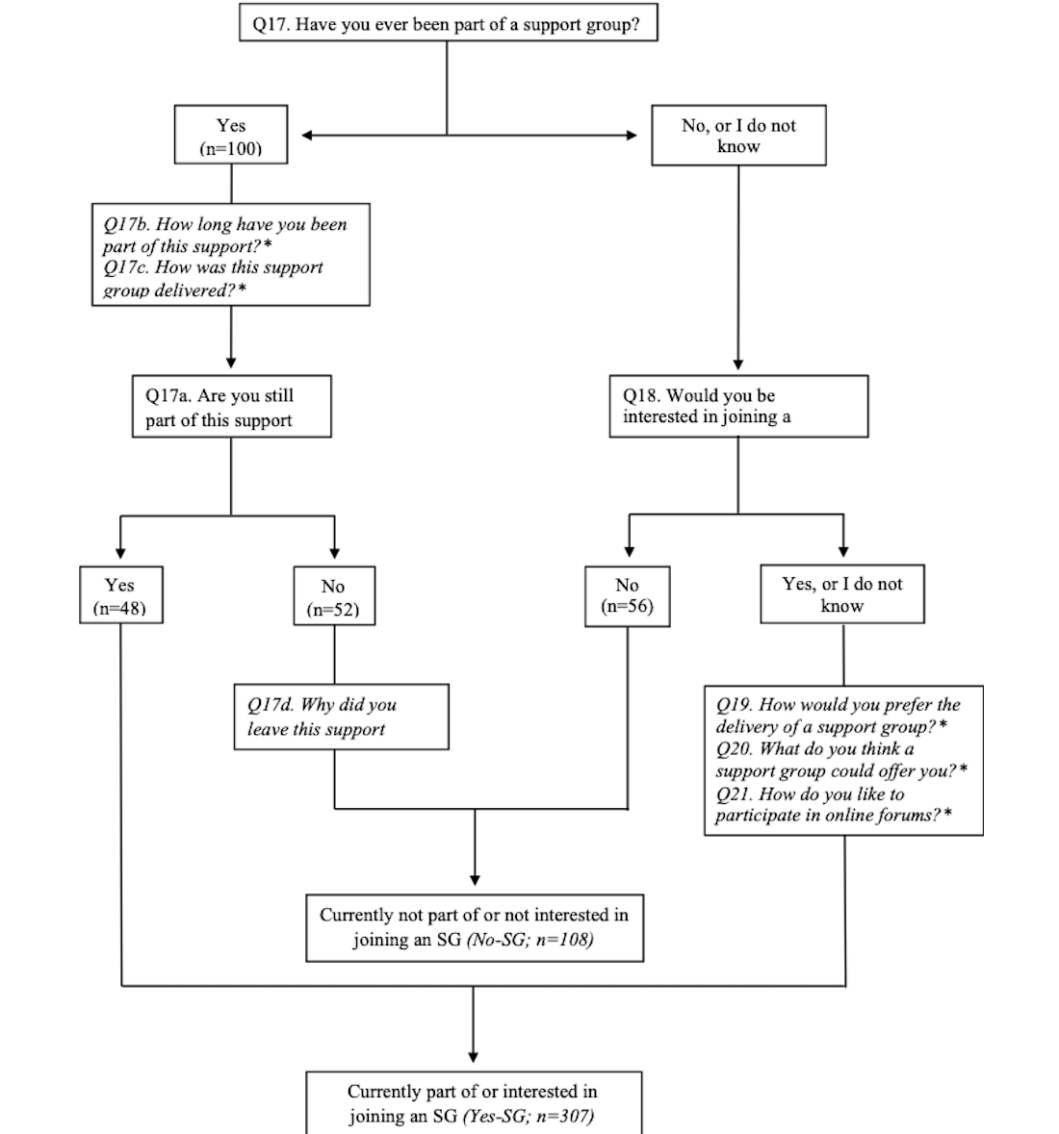
Flowchart of questions for classifying respondents into Yes-SG and No-SG. Logic questions included in the survey that were not used for classification are specified with an asterisk. Q: question; SG: support group.

All data were exported from REDCap into Microsoft Excel, and quantitative data were processed using the Statistical Package for the Social Science (version 13.0, IBM). All nominal or categorical variables were described with absolute frequency and percentages, and ordinal data were described with median and interquartile range. Respondents were categorized in 2 groups based on their response to the question “Have you ever been a part of an SG?” (Q17), followed by the question “Are you still a part of this SG?” (Q17a). If the answer on the former (Q17) was *no*, this was followed by the question: “Would you be interested in joining an SG?” (Q18). The *Yes-SG* group were respondents that were either currently part of an SG (*Yes* to Q17 and Q17a) or interested in joining one (*No* to Q17 followed by *Yes* to Q18). The *No-SG* group were respondents that were neither currently part of an SG nor interested in joining one (*No* to Q17a and Q18; Figure 1). Group differences were assessed with Chi-square (categorical data) and Mann-Whitney U (ordinal data) tests. The significance level was set at 5%.

#### Qualitative Analysis

Data from all respondents (Yes-SG and No-SG groups) were considered in the qualitative analysis. To explore perspectives on the barriers and enablers to involvement in an OSG, inductive thematic analysis was conducted with the free-text responses following principles outlined by Braun and Clarke [27]. First, 3 health researchers familiarized themselves with the entire qualitative dataset by reading, rereading, and noting preliminary codes related to the study objective (JS, JE, and MP) [27]. Codes were then grouped into provisional themes using Microsoft Excel by a researcher (MP). Coding anomalies and provisional themes were then discussed, and themes were refined until a final theming structure was agreed upon (JS, MP, KM, and TE). A theme was considered a final theme if it captured perspectives of multiple responders and was grounded in the data. All relevant criteria of the consolidated criteria for reporting qualitative research checklist were addressed to ensure qualitative rigor [28].

## Results

### Respondents

A total of 695 respondents accessed the survey. Of these, 39 did not meet the inclusion criteria, and 235 did not complete the survey. In total, 415 respondents with OA completed the survey and were included in the analysis. The Yes-SG group comprised those who were either currently part of an SG (n=48) or interested in joining one (n=259). The No-SG group comprised those who were neither currently part of an SG (n=52) nor interested in joining one (n=56; Figure 1).

### Quantitative Results

#### Sociodemographic Characteristics and Health Care– and Health Information–Seeking Behavior

Sociodemographic characteristics and health care– and health information–seeking behavior of respondents are described in Table 1. The majority of respondents were female (300/415, 72.3%) and lived in a major city (252/415, 61.0%). Employment status revealed that 189 out of 415 respondents were retired (189/415, 45.8%), 165 were working (165/415, 40.0%), 31 were on a pension (other than age pension; 31/415, 7.5%), and 28 were not working (eg, unemployed or caring for another person; 28/415, 6.8%). Technology and media (eg, internet searches, social media, newspaper, or television) were used for health information seeking by the majority of respondents (367/415, 88.4%). Sociodemographic characteristics and current health care– or health information–seeking behavior were not significantly different between Yes-SG and No-SG respondents.

**Table 1:** Sociodemographic characteristics and health care– and health information–seeking behavior of the survey respondents.

Sociodemographic characteristics and health information–seeking behavior		All respondents (N=415)	Yes-SG^a^ (N=307)	No-SG^b^ (N=108)	*P* value
Sex (female), Q^c^3, n (%)		300 (72.3)	225 (73.3)	75 (69.4)	.44
**Accessibility/Remoteness Index of Australia code^d^ (Q3), n (%)**					.62
	Major city	252 (61.0)	184 (60.3)	68 (63.0)	
	Inner regional	110 (26.6)	88 (28.9)	22 (20.4)	
	Outer regional	42 (10.2)	30 (9.8)	12 (11.1)	
	Remote	9 (2.2)	3 (1.0)	6 (5.6)	
**State of residence (Q3), n (%)**					.02
	Australian Capital Territory	19 (4.6)	16 (5.2)	3 (2.8)	
	New South Wales	289 (69.6)	214 (69.7)	75 (69.4)	
	Queensland	28 (6.7)	22 (7.2)	6 (5.6)	
	South Australia	6 (1.4)	3 (1.0)	3 (2.8)	
	Tasmania	9 (2.2)	6 (2.0)	3 (2.8)	
	Victoria	54 (13.0)	40 (13.0)	14 (13.0)	
	Western Australia	10 (2.4)	6 (2.0)	4 (3.7)	
**Employment (Q5), n (%)**					.09
	Retired	189 (45.8)	145 (47.5)	44 (40.7)	
	Working	165 (40.0)	115 (37.7)	50 (46.3)	
	Pension	31 (7.5)	27 (8.9)	4 (3.7)	
	Not working (eg, unemployed or caring for another person)	28 (6.8)	18 (5.9)	10 (9.3)	
**Financial status (Q6), n (%)**					.65
	Careful	179 (43.1)	132 (43.0)	47 (43.5)	
	Able to manage	32 (7.7)	21 (6.8)	11 (10.2)	
	Straining	133 (32.0)	99 (32.2)	34 (31.5)	
	Comfortable	71 (17.1)	55 (17.9)	16 (14.8)	
**Education (Q7), n (%)**					.90
	Year 11 or below	67 (16.2)	46 (15.0)	21 (19.4)	
	Year 12	28 (6.8)	22 (7.2)	6 (5.6)	
	Certificate 3 or 4	57 (13.8)	41 (13.4)	16 (14.8)	
	Diploma/advanced diploma	92 (22.2)	69 (22.5)	23 (21.3)	
	Undergraduate	79 (19.1)	60 (19.6)	19 (17.6)	
	Postgraduate	91 (22.0)	68 (22.2)	23 (21.3)	
Limitation of daily activities (0-100), Q8, median (IQR)		52 (31-66)	52 (31-66)	51 (32.5-65)	.92
**Seeking professional health care (Q9), n (%)**					.57
	I do not currently	100 (24.1)	75 (24.4)	25 (23.1)	
	Once a year	36 (8.7)	23 (7.5)	13 (12.0)	
	Once every 6 months	50 (12.0)	39 (12.7)	11 (10.2)	
	Once every 3 months	85 (20.5)	63 (20.5)	22 (20.4)	
	Once monthly	108 (26.0)	83 (27.0)	25 (23.1)	
	Once weekly	36 (8.7)	24 (7.8)	12 (11.1)	
**Use of technology for health information seeking (Q11), n (%)**					.38
	Yes	367 (88.4)	274 (89.3)	93 (86.1)	
	No	48 (11.6)	33 (10.7)	15 (13.9)	
**Types of technology used (ranked in top 3 for Q12), n (%)**					—^e^
	Website endorsed by advocacy group	228 (62.1)	177 (64.6)	51 (54.8)	
	Google or internet search	172 (46.9)	132 (48.2)	40 (43.0)	
	Health app	143 (39.0)	111 (40.5)	32 (34.4)	
	Wikipedia	117 (31.9)	97 (35.4)	20 (21.5)	
	Newspaper/magazine	87 (23.7)	58 (21.2)	29 (31.2)	
	Free flyers	81 (22.1)	50 (18.3)	31 (33.3)	
	Internet forums	70 (19.1)	49 (17.9)	21 (22.6)	
	Podcasts	69 (18.8)	50 (18.3)	19 (20.4)	
	Television/radio	68 (18.5)	48 (17.5)	20 (21.5)	
	Social media	66 (18.0)	50 (18.3)	16 (17.2)	

^a^Using or wishing to join a support group.

^b^Not using and not interested in joining or using a support group.

^c^Q: question.

^d^Australian International Standard Recording Code national agency.

^e^Not applicable.

#### Digital Literacy

Digital literacy characteristics, including the type of electronic device, frequency of internet use, and self-reported ability to use the internet, were not statistically different (*P*<.05) between Yes-SG and No-SG groups. Respondents reported that they used all types of devices (mobiles, tablets, laptops, and desktop computers). The majority of respondents (334/415, 80.5%) indicated accessing the internet every day, and 351 out of 415 respondents rated themselves as having good or excellent ability to use the internet (85.4%; Multimedia Appendix 3).

#### Participation and Preferences of Support Groups

For those who reported having been part of an SG (N=100), 32 had been part of it for <6 months, 28 between 6 months and 2 years, and 40 for >2 years. The majority participated in an SG delivered in person (54/100, 54.0%) or Web-based through social media (eg, Facebook; 41/100, 41.0%). Remaining respondents (3/100, 3%) participated over the phone and Web-based through a specialist website. For those who were not currently part of an SG (N=52), only 29 informed the reasons for leaving it. The main reported reason was “Not enough time to participate” (18/29, 62.1%), followed by “I did not find the information relevant to me” (6/29, 20.7%) and “I did not agree with the information on the SG” (3/29, 10.3%).

**Figure 2:**
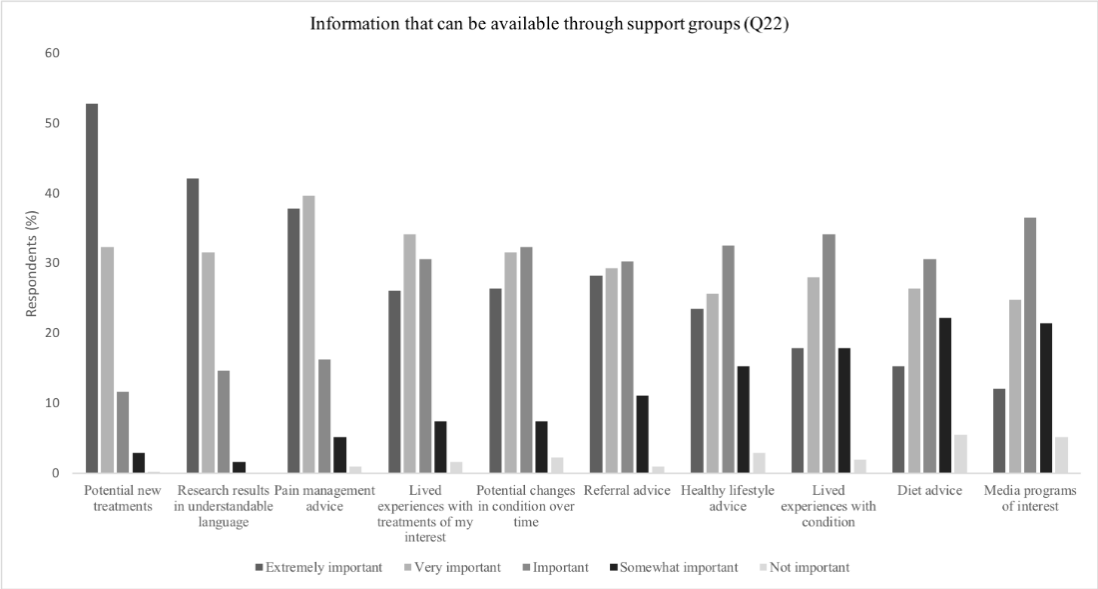
Preferences on information distribution for Yes–support group.

**Figure 3:**
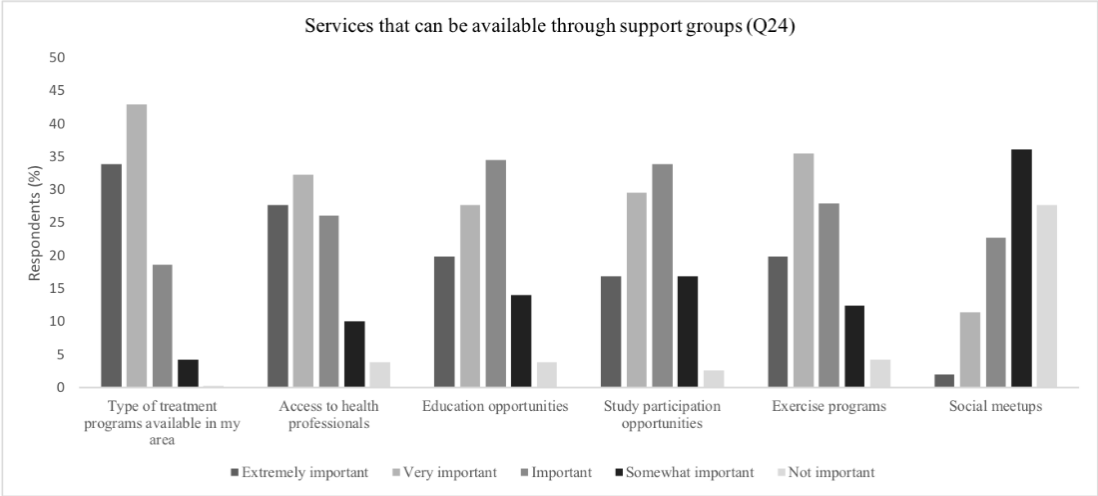
Preferences on service distribution for Yes–support group.

Regarding the level of importance of the different types of information that could be provided, Yes-SG respondents most frequently reported information pertaining to having *research results explained in language that was understandable*, *potential new treatments*, and *pain management advice* as being (extremely/very) important (302/307, 98.4%; 297/307, 96.7%; 288/307, 93.8%; respectively). However, *diet advice* and a discussion on *media programs of interest* were selected least often (Figures 2 and 3). For the types of services that could be available through SGs, respondents selected having *treatment programs available in my area* and *access to health professionals* as (extremely/very) important (293/307, 95.4%; and 264/307, 86.0%; respectively), whereas having *social meetups* was selected least often (111/307, 36.2%).

Among all respondents, 369 out of 415 (88.9%) thought they would (strongly) benefit from an OSG, 260 out of 415 (62.7%) thought receiving support from peers is (extremely) important, and 243 out of 415 (58.6%) were (extremely) motivated to use an OSG. Within the Yes-SG group, 126 out of 259 respondents (48.7%) indicated that they would prefer to access an SG online (eg, online format), 67 (25.9%) through a face-to-face meeting, 58 (22.4%) via email, and 8 (2.9%) via phone (Multimedia Appendix 2). Of those preferring OSG (N=126), 31.7% (40/126) would mainly participate in an OSG by *commenting, discussing, or debating topics*, and 31.7% (40/126) indicated they would prefer to *only read articles*. Remaining responses included *asking questions* (20/126, 15.9%), *sharing articles from the OSG with non-OSG members* (16/126, 12.7%), and *having direct contact with a moderator* (4/126, 3.2%). Respondents within the Yes-SG group indicated a high level of trust (average level of trust 73.4/100 points) in advice provided by a health professional. Interestingly, trust in information provided by a trained peer facilitator with the same condition was equally high (71.7/100 points).

### Qualitative Results

Thematic analysis of the qualitative responses identified 5 key themes related to barriers and enablers to OSG use: (1) ease of access, (2) enjoyment of experience, (3) information quality, (4) time, and (5) motivation. An overview of themes and subthemes is provided in Multimedia Appendix 4. Respondents are distinguished by numbers where quotes are used.

#### Theme 1: Ease of Access

Analysis identified that *ease of access* was a key concern for respondents when considering using an OSG for OA. Some respondents noted various technological factors that would help them access the OSG. These factors included making sure that the OSG was accessible to people of all abilities. Respondents identified accessibility features such as larger fonts, subtitles, clear sound and visuals, voice-activated programs, and the ability to save and print content. Many respondents emphasized a preference for an intuitive design, including making the OSG easy to use with minimal passwords, clear step-by-step instructions, technical support, and compatibility across browsers. For example, there were suggestions for *a well-structured webpage that is easy to search* (Participant 1) and *an OSG that is quick and easy to use* (Participant 2). Respondents also said it would be helpful if the OSG could be accessed across different devices, such as computers, laptops, and mobile phones. Variable internet availability and reliability as well as variable levels of digital skills were also frequently mentioned as important access considerations for OSGs.

#### Theme 2: Enjoyment of Experience

The *enjoyment of experience* of participating in an OSG was also identified as an important theme for respondents. Physical comfort (eg, pain, fatigue, and poor concentration ability) and personality/mood were raised as concerns by some respondents because of the potential effect of these factors on their ability to interact with an OSG. Respondents mentioned the need to encourage empathy and positivity among members of an OSG for OA and to avoid negativity and pessimism. For example, Participant 4 said:

[OSGs] can be very supportive but sometimes they seem to attract people who have had negative experiences with treatment, health professionals, etc. So, you need to be careful of some comments and information.

Participant 4

The impersonal nature of online contact was mentioned by many respondents, and having access to personalized features within the OSG, such as familiar people, face-to-face opportunities, and a contactable person for phone and/or online support, was requested. For example, Participant 5 said:

[The OSG] loses the personal touch. Like talking to a computer!! You wouldn't know if your problem is being addressed or if it’s generalized.

Participant 5

#### Theme 3: Quality of Information

Quality of information is considered an important aspect of an OSG. Respondents discussed that the content of an OSG should include relevant, novel, and dynamic information on a range of different topics that are tailored to the individual needs. For example, Participant 6 said:

Maybe specific weekly topics and activities—that would keep me more motivated.

Participant 6

In addition, respondents said that it was essential that the information provided in the OSG is trustworthy and facilitators are qualified. Participant 7 stated:

[I] would not like the sessions [within the OSG] to be just chat sessions. I believe they should be chaired by a medical specialist in the OA field.

Participant 7

Overall, respondents highlighted that information should be trustworthy and distributed in a clear and concise language that avoids jargon.

#### Theme 4: Time

The concept of *time* was mentioned by most respondents. Some respondents made assumptions that OSGs are held at set times, and in this case, they expressed concerns about the need for planning and organizing. For example, Participant 8 mentioned:

[I would prefer] a specific day and time allocated on a fortnightly or monthly basis.

Participant 8

Similarly, flexibility with regard to the amount of time to engage and the time of day seemed important to respondents. The ability to return to information at a later time or print was also suggested:

Just being able to access at any time the information.

Participant 9

Most respondents highlighted that having limited time available per day might act as a barrier to their engagement with an OSG.

#### Theme 5: Motivation

Respondents reported different views on *motivation*. Some respondents reported that they were highly motivated to try an OSG:

I am in full support of this venture, especially as I live in a regional town with minimal services and access to information comes mainly from the Internet.

Participant 10

Some reported they might require more motivation before becoming involved in an OSG:

I'm a bit skeptical, but would give it a try.

Participant 11

Others reported they lack motivation:

I don’t really like online anything.

Participant 12

Respondents suggested that reminders and notifications via SMS and/or email may facilitate engagement. Also, knowledge of the potential benefits of OSGs could help motivate patients to be involved.

## Discussion

### Principal Findings

This study used a mixed method design to explore health care– and health information–seeking behavior, digital literacy, preferences, and barriers for the design of SGs for people living with OA. Of the 415 survey participants, 307 (74.0%) were either currently using or wishing to join an SG, and the majority identified *online* as their preferred mode of delivery rather than via email, phone, or face-to-face. For those who were currently part of an SG, the 2 main methods of delivery were in person and Web-based (eg, social media). Most participants reported that they were currently using the internet on a range of devices to access health information. The majority felt that they would benefit from the participation in an SG and indicated trustworthy and qualified health professionals and peer leaders as preferred facilitators of SGs. Furthermore, up-to-date quality information (eg, new treatments, latest research results, and pain management advice) in lay language was deemed important. Qualitative analysis revealed a lack of time and motivation as the main barriers for OSG participation. Although from a small number of respondents, the reported reasons for leaving an SG sustain these qualitative findings. Respondents suggest that factors including information about benefits and reminders could facilitate engagement. The main enablers were related to accessibility, enjoyment of the experience, and quality (novel and trustworthy) of the information.

### Strengths and Limitations

Strengths and limitations of this study need to be considered. Strengths included the size of the respondent group (well powered to provide generalizable data) and the high response rate after distribution of the survey. However, it is important to note the limitations to the generalizability of this study that are highly contextual. As participants represent a sample of convenience, the results may not represent the views of all people with hip, knee, or back OA. Results may also not be applicable across countries, particularly, where cultural and social conditions differ considerably from the Australian context. As recruitment was undertaken via an institutional patient database, there is also the possibility that our cohort is more comfortable with, and capable of engaging with, technology. In addition, respondents were also likely to be active seekers of health information, have English language competency, and have higher health literacy. As such, participants may not represent vulnerable groups, including people who need additional support for such health engagement and those with culturally and linguistically diverse background. A limitation of the survey is that it may not reflect all types of (online) health information resources. As such, the results should be interpreted relative to the conducted survey. This study had a high representation of people who are either currently using or interested in joining an SG (Yes-SG) and may underrepresent people who do not use SGs. Furthermore, it is possible that respondents who were not currently part of an SG (part of the No-SG group) were still interested in joining another SG, but this information was not collected. The authors acknowledge potential differences in health care– and health information–seeking behavior, digital literacy, and preferences within the Yes-SG group, for example, differences between people who are currently using and those who are interested in joining an SG. Although the majority of the Yes-SG group comprised people interested in joining an SG (259/307, 84.4%), further research is required to understand if there are differences in preferences (eg, specialist website, social media, or in person) between people who are willing to use OA SGs and those who are already in such groups. The quantitative analysis examined differences between Yes-SG and No-SG for survey questions regarding SGs including OSGs, whereas the qualitative analysis included data of all respondents but only related to OSG questions. This needs to be considered when interpreting the results. Data used for the qualitative analysis of this study were obtained through 3 open-ended survey questions. This approach potentially limits the ability to conduct an in-depth exploration of individuals’ attitudes and beliefs regarding OSGs, which may be possible with interviews. However, it does enable anonymous responses, which may be advantageous by reducing the risk of a Hawthorn effect bias.

### Comparison With Previous Studies

Previous research reports that people with higher income and education levels [29,30], those living with a chronic health condition [10], and those who are more proactive in seeking health information [31] are more likely to engage in OSGs. Similarly, our survey respondents were relatively well educated; however, they also were confident using technology to seek health information, and the majority of the Yes-SG group preferred OSGs. Respondents also emphasized that it would be helpful if the OSG could be accessed across different devices (computers, laptops, and mobile phones) or as an app. However, the qualitative analysis revealed that respondents had variable levels of digital skills and indicated a preference for intuitive, simple designs with clear step-by-step instructions including technical support. This finding is in line with recommendations from previous studies that state OSGs should aim to employ simple navigation design, visually appealing sites, compatibility across multiple devices, and accessible and printable content to ensure people with all levels of computer experience can participate [32,33].

Respondents in our study rated advice on pain management, new treatment options, and provision of research results in a consumer-friendly language as extremely important when they were asked to rate different types of information that could be available via SGs. Similarly, the qualitative analysis revealed the importance of having access to relevant, novel information on a range of topics tailored to individuals in an OSG. Dynamic information is preferred in clear and concise language that avoids jargon. Previous research has indicated that accurate and up-to-date information can promote active participation, allow people to make informed choices [34], and give them greater control over their own health care decisions [35,36]. Therefore, we recommend future OSGs integrate up-to-date relevant information that is simple to follow. This is potentially implementable through the use of subject headings, keywords, or moderator-driven explanations of complex topics.

The majority of respondents who were currently using or interested in joining SGs reported that they were likely to trust advice from either a health professional or a trained peer facilitator. However, qualitative analysis highlighted that some respondents felt the opposite. Specifically, respondents stressed the importance of attaining trustworthy information from online facilitators in OSGs. Previous research examining enablers and barriers to using SGs in patients with arthritis, breast cancer, or fibromyalgia found that older participants (compared with younger ones) did not favor OSGs because of a lack of trust in the internet [37]. Our participants were all aged >45 years and might have held similar perceptions regarding distrust of information from the internet. Trust in the OSGs might be facilitated by several strategies, such as embedding the group within trusted host sites (eg, consumer advocate organizations), use of a health professional or trained peer moderator or maintaining a minimum number of group membership to permit diversity of opinion. It is currently unknown whether a strategy is more effective than another at increasing consumer trust.

### Conclusions

From this study, we suggest that the use of SGs could be facilitated by the inclusion of digital options such as email, social media, and health websites to enhance engagement. Our findings also suggest that efforts need to be made to ensure the online platform is intuitive and accessible. Information to help people make decisions about which treatments to seek are desired by users of OSG. Other important features of an OSG for hip/knee OA or back pain include having an expert health professional or trained peer facilitator to moderate the OSG, providing information that is free of jargon, and incorporating reminders to facilitate engagement. Members also need to feel confident about the security of their personal information, the trustworthiness of the information and advice, and the credibility of the experts providing input to the group. Finally, a moderator or facilitator’s role should include efforts to maintain interest, so the membership continues to be motivated to engage.

## Appendix

Multimedia Appendix 1 Checklist for Reporting Result of Internet E-surveys (CHERRIES).

Multimedia Appendix 2 Survey questions.

Multimedia Appendix 3 Table S1. Digital literacy characteristics of survey respondents.

Multimedia Appendix 4 Table S2. Themes and subthemes derived from the qualitative analysis.

## Abbreviations

CCSMS: Chronic Condition Self-Management Support
HREC: Human Research Ethics Committee
NHMRC: National Health and Medical Research Council
OA: osteoarthritis
OSG: online support group
REDCap: Research Electronic Data Capture
SG: support group
