# Supplementary material for: Exploring the Characteristics and Preferences for Online Support Groups: Mixed Method Study
Source: J Med Internet Res. 2019 Dec 3;21(12):e15987. doi: 10.2196/15987 (PMC6918205; doi:10.2196/15987)
Supplement: Multimedia Appendix 1 [file jmir_v21i12e15987_app1.docx]

Multimedia Appendix 1. Checklist for Reporting Result of Internet E-surveys (CHERRIES).

| **Item category** | **Checklist Item** | **Explanation** |
| --- | --- | --- |
| Design | Describe survey design | The survey was targeted to people who had previously been clinically diagnosed with osteoarthritis (of any joint) and who had registered their interest in participating in webinars, research and receiving relevant OA-specific information on a databased hosted by a bone and joint research institute. All participants were required to be older than 45 years of age and experiencing pain in their back, hips or knees for the past 3 months. |
| IRB approval and informed consent process | IRB approval | Ethics approval was obtained from the Human Research Ethics Committee of the University of Sydney (HREC #2017/957). |
|  | Informed consent | The landing page of the survey was a participant information statement. This statement provided details on (a) the purpose of the study (b) the duration of the survey (minutes to completion) (c) the investigators involved with the study (d) specific requirements of the survey (e) risks and benefits of participating and (f) how to register complaints or concerns about the study with an independent third party (the University ethics committee). Participants consented by ticking a box at the bottom of the statement. |
|  | Data protection | No identifiable personal data was collected. |
| Development and pre-testing | Development and testing | The survey was developed by the researchers involved in the study. Questions pertaining to respondents’ healthcare behaviour and use of technology was piloted with a small sample of the target population to ensure usability and validity of the questions. Technical aspects of the study i.e. the logical flow of the survey was tested by the research team prior to dissemination. |
| Recruitment process and description of the sample having access to the questionnaire | Open survey versus closed survey | The survey was open design. |
|  | Contact mode | Initial contact with potential respondents was made through the internet. |
|  | Advertising the survey | The survey was advertised via generic email invitation sent to all database members who had indicated they were interested in participating in future research. A link to the survey was also posted on the Research Institute’s social media site. |
| Survey administration | Web/email | The survey was administered through the Research Electronic Data Capture (REDCap) survey software. |
|  | Context | REDCap is a purpose designed software for survey capture used primarily by research institutes and universities. Each survey built within the software has a unique link/address. In order to meet ethical requirements of these institutes, the surveys cannot be accessed through generic websites but can be freely accessed (i.e. open accessed) by anyone who has the link. |
|  | Mandatory/voluntary | The survey was voluntary. Respondents could also cease answering questions at any point. |
|  | Incentives | There were no incentives offered. |
|  | Time/Date | Data collection occurred between March 2018 and September 2018. |
|  | Randomization of items or questions | Questions were not randomised. |
|  | Adaptive questioning | An adaptive logic was present throughout the survey in order to reduce the response burden for respondents. |
|  | Number of items | The maximum number of questions that a respondent may have been presented with was 38. As the survey software scales the number of questions per page based on the devise being used (i.e. smart phone versus tablet versus computer screen), the number of items per page could vary from 2 to 8. |
|  | Number of screens | This would have been based on the number of items per screen, which was dependent on the device the respondent used to access the survey. The maximum number of screens would have been approximately 16. |
|  | Completeness check | Due to institutional ethical review board requirements, response selection was not enforced for questions regarding healthcare behaviour, technology use or support groups. All submitted responses were analysed regardless of whether the entire survey was complete or not. |
|  | Review step | The survey had a “back” button enabled respondents to review previously answered questions at any time prior to final submission. |
| Response rate | Unique site visitor | Each submission was identified with a unique response code. |
|  | View rate | View rate was not quantified. |
|  | Participation rate | 59.4% of people visiting the survey agreed to participate. |
|  | Completion rate | All participants who agreed to participate submitted the survey, although not everyone who submitted the survey responded to every question. |
| Preventing multiple entries from the same individual | Cookies used, IP check | REDCap does not use cookies or IP check. |
|  | Log file analysis | The demographics of all submitted responses were analysed to identify possible duplicate entries. This included a check of postcodes, gender, income and age. |
| Analysis | Handling of incomplete questionnaires | All submitted responses were analysed regardless of whether the entire survey was complete or not. |
|  | Questionnaires submitted with an atypical timestamp | We did not measure how long respondents took to complete the survey. |
|  | Statistical correction | No weighting or propensity scores were placed on questions. |
