# Supplementary material for: Exploring the Characteristics and Preferences for Online Support Groups: Mixed Method Study
Source: J Med Internet Res. 2019 Dec 3;21(12):e15987. doi: 10.2196/15987 (PMC6918205; doi:10.2196/15987)
Supplement: Multimedia Appendix 2 [file jmir_v21i12e15987_app2.docx]

Multimedia Appendix 2. Survey questions.

Block 1: Sociodemographic characteristics

1. Are you over 45?

● Yes

● No

Logic: No – exit survey

2. Do you have knee, hip or back pain lasting more than 3 months?

● Yes

● No

Logic: No – exit survey

3. What is your postcode

Open answer

4. What sex do you identify as?

● Female

● Male

● Other

● I choose not to identify

5. Which of the following best describes your current employment status?

● Working full time

● Working part time

● Retired

● Domestic duties

● Unemployed

● Carer for another person

● Government Benefits/Pension

● I would prefer not to answer this question

6. How would you describe your financial situation?

● Find it a strain to get by from week to week

● Have to be careful with money

● Able to manage without much difficulty

● Quite comfortably off

● Very comfortably off

7. What is the highest level of education you have completed?

● No schooling

● Primary/elementary school only

● Year 11 or below

● Year 12 (Final year of high school/college)

● Certificate III or IV

● Diploma/Advanced Diploma

● University undergraduate degree

● Postgraduate degree

Block 2: Health and Information-seeking Behaviour

8. Does your condition limit your ability to perform daily tasks?

Scale: Not at all – completely incapacitated.

9. Are you currently seeking care from a health professional for your knee, hip or back pain?

● Once weekly

● Once monthly

● Once every 3 months

● Once every 6 months

● Once a year

● I do not currently seek care from a health professional

10. Do you seek health information from any of the following? (Check all that apply)

● GP (family doctor)

● Sports physician

● Orthopaedic surgeon

● Rheumatologist

● Physiotherapist

● Dietician

● Exercise Physiologist

● Podiatrist

● Osteopath

● Chiropractor

● Naturopath

● Gym/fitness instructor/personal trainer

● Nurse [ST]

● Other: please specify [ST]

11. Do you ever use technology or media for health information (e.g. internet searches, social media, newspaper, TV)?

● Yes

● No

● I don’t know

12. Logic: if 11 is yes

Rank the following technology and media in the order that you prefer to use them to gain health information (1 = would like to use most)

● Health App

● Website endorsed by advocacy group e.g. Arthritis Australia

● Wikipedia

● Google/Internet search

● Social Media

● Internet forums

● Podcasts

● TV/radio

● Newspaper/magazine

● Free flyers

Block 3: Use of Technology

13. Which of your following devices do you use for internet access?

● Mobile phone drop down: yes, no, I do not own this device

● Hand-held tablet/iPad drop down: yes, no, I do not own this device

● Laptop/notebook computer drop down: yes, no, I do not own this device

● Desktop computer drop down: yes, no, I do not own this device

14. How often do you use the internet (including browsing information, email and other online activities, and social media like Skype etc)? (please choose one)

● Never

● Once every few months

● Once a month

● Once a week

● Several times a week

● Everyday

15. Logic: skip if “Never” is ticked in 14

How would you rate your ability to use the internet (including browsing information, email and other online activities and social media like Skype etc.)? (please choose one)

● Poor

● Fair

● Good

● Excellent

Block 4: Support Groups

16. Has your health professional ever suggested you join a support group for your condition?

● Yes

● No

● I don’t know

17. Have you ever been part of a support group?

● Yes

● No

● I don’t know

Logic if yes

17a. Are you still part of this support group?

● Yes

● No

Logic if 17a yes

17b. How long have you been part of this support?

● 1 to 3 months

● 3 to 6 months

● 6 to 12 months

● 1 to 2 years

● 2 to 5 years

● More than 5 years

Logic if 17a is Yes

17c. How was this support group delivered?

● Online through social media (e.g Facebook)

● Online through a specialist website

● In person (e.g., monthly meetings)

● Over the phone

● Other please specify

Logic if 17a is No

17d. Why did you leave this support group?

● Not enough time to participate

● I did not find the information relevant to me

● I did not agree with the information on the support group

● I found the language or conversation offensive

● I found the information too difficult to understand and apply to my life

● I did not feel that my opinions were valued

● I had an unreliable internet and could not participate fully

● There was not enough moderation of content by an expert

● There was too much moderation from experts

● I did not like the frequency of posts (too much or too little)

● Other please specify

Logic: display if 17 is “no” or “I don’t know”

18. Would you be interested in joining a support group?

● Yes

● No

● I don’t know

Logic: only display the following if 18 IS NOT responded “No”

19. How would you prefer the delivery of a support group?

● Online

● In-person meeting

● Over the phone

● Via email

● Other please specify

20. What do you think a support group could offer you?

● Motivation

● Knowledge about your condition

● Social Support

● Resources

● Referrals to further healthcare sources

● Ideas about how you can manage your condition

● Other please specify

21. How do you like to participate in online forums?

Logic: only offer if “online” is ticked in 19

● Read articles only

● Share articles from the forum to other people or platforms

● Post information that I think may help others on the forum

● Comment on articles or information from the forum

● Comment on questions or opinions that other forum members have expressed

● Discuss or debate issues with other members of the forum

● Ask question on forum

● Have direct contact with the moderator

● Other please specify

22. How important is it for you that the following information are available through support groups?

From not important at all to very important

● How my condition might change over time

● Pain management advice

● Diet advice

● Potential new treatments

● Other peoples’ experiences with the condition that I have

● Other people’s experiences with treatments that I am interested in

● Results from new scientific research presented in a way that is understandable

● Media programs about my condition

● Advice regarding who I could be referred to

● Healthy lifestyle advice

23. Are there any other types of information that you would like to have available through a support group?

Free text

24. How important is it for you that the following services are available through support groups?

● Access to health professionals

● Social meet-ups

● Exercise programs

● Access to opportunities to participate in studies

● Access to educational opportunities (webinars, community events, summits, etc.)

● What type of treatment programs might be available in my area

25. Are there any other services that you would like to have available through a support group?

Free text

26. How likely are you to trust the advice of a health professional facilitating an online forum?

Not at all likely ------------------------------------------------------------Definitely likely

27. How likely are you to trust the advice of a trained person with the same condition as you facilitating an online forum?

Not at all likely ------------------------------------------------------------Definitely likely

28. How comfortable are you to ask a health professional facilitating a forum questions related to your problem?

Not at all likely ------------------------------------------------------------Definitely likely

29. How comfortable are you to ask a trained person with the same condition as you facilitating an online forum questions related to your problem?

Not at all likely ------------------------------------------------------------Definitely likely

30. Do you think you will benefit from using the Online Support Group (OSG)?

a. No / I will benefit from it / I will strongly benefit from it

31. How important is it for your OA management to receive support from a group of people that suffer from the same condition as you?

a. Not Important at all/ Neither Important nor Unimportant / Important / extremely important

32. How motivated are you to use an OSG?

a. Not motivated at all / Neither motivated nor unmotivated / Motivated / Extremely motivated

33. How do you feel about using the OSG on your device (mobile, tablet, computer)?

a. Multiple choice – Not looking forward to it / Sceptical / Stressed and/or Anxious / Neutral / Enthusiastic

34. How confident are you that you will be able to use an OSG on your device (mobile, tablet, computer)?

a. Not confident at all / Neither confident nor not confident / Confident / Extremely confident

35. How capable are you to use the OSG on your device (mobile, tablet, computer)?

a. Not capable at all / Neither capable nor incapable / Capable / Extremely capable

36. What would make it difficult for you to use the OSG?

Free text

37. What would make it easier for you to use the OSG?

Free text

38. Is there anything else you would like to say about using online support group?

Free text
