# Supplementary material for: Exploring the Characteristics and Preferences for Online Support Groups: Mixed Method Study
Source: J Med Internet Res. 2019 Dec 3;21(12):e15987. doi: 10.2196/15987 (PMC6918205; doi:10.2196/15987)
Supplement: Multimedia Appendix 3 [file jmir_v21i12e15987_app3.docx]

Multimedia Appendix 3. Table S1. Digital literacy characteristics of survey respondents. Data presented with absolute frequency (percentage, %).

|  | All respondents  (n = 415) | Yes-SG  (n = 307) | No-SG  (n = 108) | *P*-value |
| --- | --- | --- | --- | --- |
| Use of devices for Internet access (Q13)  Mobile  Tablet  Laptop  Desktop computer | 325 (78.3)  240 (57.8)  281 (67.7)  231 (55.7) | 236 (76.9)  181 (59)  208 (67.8)  166 (54.1) | 89 (82.4)  59 (54.6)  73 (67.6)  65 (60.2) | 0.23  0.43  0.98  0.27 |
| Frequency of Internet use (Q14)  Every day  Several times a week  Once a week  Once a month | 334 (80.5)  54 (13)  12 (2.9)  15 (3.6) | 246 (80.1)  42 (13.7)  7 (2.3)  12 (3.9) | 88 (81.5)  12 (11.1)  5 (4.6)  3 (2.8) | 0.53 |
| Ability to use Internet (Q15)  Excellent  Good  Fair | 155 (37.7)  196 (47.7)  60 (14.6) | 115 (38)  150 (49.5)  38 (12.5) | 40 (37)  46 (42.6)  22 (20.4) | 0.13 |

Abbreviations: Yes-SG: using or wishing to join a support group; No-SG: not interested in joining or using a support group.
