# Supplementary material for: Exploring the Characteristics and Preferences for Online Support Groups: Mixed Method Study
Source: J Med Internet Res. 2019 Dec 3;21(12):e15987. doi: 10.2196/15987 (PMC6918205; doi:10.2196/15987)
Supplement: Multimedia Appendix 4 [file jmir_v21i12e15987_app4.docx]

Multimedia Appendix 4. Table S2. Themes and subthemes derived from the qualitative analysis.

| Themes | Subthemes | | | | |
| --- | --- | --- | --- | --- | --- |
| Ease of access | Assistive technologies & accessibility | Cross-platform compatibility | Internet access | Intuitiveness | Technical proficiency |
| Enjoyment of experience | Personality/mood | Physical comfort | Reduce difficult personalities, needy people, negativity | Impersonal nature of the Internet |  |
| Information quality | Interesting content | Trustworthiness | Language |  |  |
| Times | Incorrect assumptions about time | Emphasise time flexibility | People are time-poor |  |  |
| Motivation | Reminders/notifications | Highlight potential benefits | Motivation needed |  |  |
